# A highly efficient auxin-producing bacterial strain and its effect on plant growth

**DOI:** 10.1186/s43141-021-00252-w

**Published:** 2021-12-02

**Authors:** Seunghye Park, A-Leum Kim, Yoon-Kyung Hong, Ji-Hwan Shin, Se-Hwan Joo

**Affiliations:** Research & Development Center, Cosmicgreen Inc., Daeryung Post Tower I, Digital-ro 288, Seoul, 08377 Republic of Korea

**Keywords:** Auxin, Plant root growth promotion, Plant-microbial interaction

## Abstract

**Background:**

Various bacteria promote plant root growth in the rhizosphere, as a measure of securing and enlarging their ecological niche. These interactions are mediated by plant growth regulators (PGRs) such as auxin, and indole-3-acetic acid (IAA) is one of the physiologically active auxin. In this study, we isolated an unusual bacterial strain from food process waste with high efficiency and demonstrated its effects on plant rooting and early-stage growth.

**Results:**

The efficiency of this bacterial strain in producing IAA was 16.6 mg/L/h in Luria-Bertani broth containing 0.05% l-tryptophan (Trp) at room temperature (24 ± 2 °C). Its IAA production was highly dependent on the presence of precursor, Trp. This bacterium was identified as *Ignatzschineria* sp. by 16S rDNA sequencing. Its bacterial culture supernatant (BCS) enhanced plant root initiation, root growth, and plant growth in the early stages. The root mass formed BCS-treated in apple mint cuttings was twofold of that formed in the control. The root number and length were 46% and 18% higher, respectively, in BCS-treated chrysanthemum cuttings than in the control.

**Conclusions:**

These results show that the BCS of *Ignatzschineria* sp. CG20001 isolate obtained in this study can be used for agricultural applications. In addition, the novelty of this strain makes it a valuable genetic resource for biotechnological applications.

**Supplementary Information:**

The online version contains supplementary material available at 10.1186/s43141-021-00252-w.

## Background

The rhizosphere is a complex environment in which plants and various types of microbes interact with each other. The interaction between plants and microbes can be beneficial or harmful, depending on the species or strains, and the internal and external conditions of each plant. Plant growth-promoting bacteria (PGPB) usually have beneficial effects on plants. The mechanisms underlying their beneficial effect can be divided into two categories: direct facilitation of plant growth and indirect influences by decreasing inhibitory effects [1]. The direct plant growth promotion mechanism includes facilitating plant nutrient acquisition and modulation of plant hormone levels. The indirect mechanism includes the suppression of plant pathogens through producing antifungal substances. In desirable cases, one bacterial strain can have both effects [2].

Direct mechanisms also include the production of plant hormones that enhance plant growth or enzymes that help with the solubilization and absorption of nutrients to plant roots. PGPB in the rhizosphere are plant growth-promoting rhizobacteria (PGPR), and many of them have been reported to produce auxin, a root growth-promoting hormone. Auxins produced by PGPR, enhance plant root growth, enabling microbes to secure and enlarge their occupancy and food supply [3, 4]. Since improved root growth also has positive effects on plant growth, many efforts have been made to discover auxin-producing capacity in rhizosbacteria. Many previous studies have reported various types of bacterial species with this ability, such as *Azospirillum* [5], *Agrobacterium* [6], *Bacillus* [7], *Bradyrhizobium* [8], *Enterobacter* [9], *Erwinia* [10], *Pseudomonas* [11], and *Rhizobium* [12]. Other bacteria can stimulate plants to produce more auxins [13].

The versatile signal transmitter, auxin, is induced and synthesized through many different pathways. Therefore, the conditions and inducers for the optimal productivity of auxin and its related metabolites vary from species to species [14]. However, plant-microbial interactions and microbial life cycles are markedly more complicated than direct relationships, and some bacteria from unusual origins, such as animal intestines, have also been found to produce auxin [15]. Surprisingly, some PGPB were isolated from unexpected sources, do not normally colonize plants [2].

Despite the various characteristics of IAA production patterns, their biosynthetic pathways share some commonalities, such as the precursor l-tryptophan. At least four different pathways use Trp as the precursor, and three of these pathways share the first step between both plants and bacteria (Fig. S1). The IAA biosynthetic pathways have been suitably explained in previous reviews [14, 16]; hence, many researchers have reported Trp as a critical inducer of IAA production.

Auxin has been reported to improve crop growth and productivity by enhancing root growth [17, 18]. In addition, auxin can improve biotic and abiotic stress tolerance in plants [19]. Therefore, microbes that produce auxin have been investigated as a biological resource for agricultural applications.

In this study, we isolated a bacterial strain from food process waste and found that this strain produced IAA with high efficiency compared to previously reported strains; therefore, we investigated its effects on plant growth. Its high efficiency of auxin production makes this bacterium a promising strain for agricultural and biotechnological use at an industrial scale.

## Methods

### Bacterial strain isolation from food process waste

Food process waste obtained from a food processing factory was resuspended in sterilized water to make 10% dilution, following which further dilutions (1:100, 1:1000) were also prepared. The diluents were spread onto a Luria-Bertani (LB) (Duchefa, The Netherlands) agar plate and incubated at room temperature for 3 days. Single colonies that appeared were randomly picked with sterilized toothpicks and then transferred to two new LB agar plates to make duplicate plates. One of the two plates was stained with Salkowski reagent, and the colonies that turned pale pink were selected for further analysis. After growing them in LB broth, the clone with the most intense red staining was selected and subjected to identification and further study.

### Identification of the bacterial strain

Genomic DNA of the isolated and cultured bacteria was isolated using a DNA isolation kit (Exgene^TM^ Cell SV mini, Ecocell, Korea). The purified genomic DNA was used as a template for 16S rDNA amplification using primers, 27F (5′-AGAGTTTGATCMTGGCTCAG-3′) and 1492R (5′-GGTTACCTTGTTACGACTTC-3′). The amplified PCR product was purified using a purification kit (Expin^TM^ Combo GP, Ecocell, Korea). The sequence was analyzed using the Sanger method by a sequencing service provider (Cosmogenetech, Korea), and the sequence similarity was analyzed through BLAST in the US National Center for Biotechnology Information (NCBI) database. For further analysis, other sequences of close bacteria were downloaded from the GenBank database to perform multiple alignments using ClustalX2 [20]. A phylogenetic tree was constructed using the neighbor-joining method [21] based on the obtained 16S rDNA sequences. The bootstrap values of 500 replications were shown next to the branches [22]. Tree construction and bootstrap tests were conducted in MEGA X [23].

### Bacterial growth and IAA content analysis

For optimization of IAA production, carbon sources and tryptophan concentration were examined. LB broth supplemented with carbon sources or tryptophan was used as a basal medium to optimize IAA production [24–26]. To analyze IAA content produced by the bacteria, *Ignatzschineria* sp. CG20001 strain was cultured in LB broth with shaking at 150 rpm, at room temperature. For the analysis of medium components, 5 g/L of glucose (BioWorld, USA) and Trp (Duchefa, The Netherlands) solutions were prepared and filtered, and a 50 g/L soluble starch (Duksan, Republic of Korea) solution was prepared and autoclaved. At several time points during the culture, the bacterial cultures were collected and centrifuged for 1 min at 13,000 rpm. The supernatants from the centrifuged cultures were subjected to colorimetric IAA analysis.

Colorimetric analysis of IAA concentration was performed using Salkowski reagent. Salkowski reagent was prepared by diluting 1 mL of 0.5 M FeCl_3_ and 24.5 mL of 70% perchloric acid in 24.5 mL distilled water to make a final concentration of 10 mM and 35%, respectively [27]. Two hundred microliters of BCS or its dilutions were mixed with 300 μL of Salkowski reagent and kept in the dark for at least 30 min at room temperature. The absorbance of the mixtures was measured at 536 nm using a spectrophotometer (UV/VIS Excellence UV5, Metler-Toledo, Switzerland). For quantitative analysis, serial dilutions from 0 to 50 mg/L of the IAA (Duchefa, The Netherlands) solution were used as the standard by dissolving IAA powder in methanol.

### LC-MS/MS analysis

To confirm the presence of IAA in the bacterial culture supernatants, the culture supernatants were freeze-dried and solubilized in methanol prior to LC-MS/MS analysis. LC-MS/MS analysis was performed using an Ultimate 3000 RS-Q-Exactive Orbitrap Plus (Thermo Scientific, USA) equipped with a 100 mm × 2.1 mm, 1.7 μm of C18 column (Aquity UPLC BEH C18). The mobile phase was 0.1% formic acid and acetonitrile in water at a flow rate of 0.4 mL/min. Five microliters of the injected samples were monitored through multiple reaction monitoring (MRM) in the positive-ion mode. IAA standard was prepared as mentioned in the above section.

### Rooting and plant growth experiments

For the rooting experiment, *Chrysanthemum morifolium* (chrysanthemum) and *Mentha suaveolans* (apple mint) plants were purchased from a local market. Six plants of each type were grown in a growth room with environmental control of air temperature 22 ± 2/18 ± 2 °C (light/dark), relative humidity of 60 ± 10%, photoperiod period of 16 h per day, and photosynthetic photon flux density (PPFD) of 180 ± 10 μmol·m^−2^·s^−1^ irradiated by fluorescent lamps (TL5 14W/865 Philips, Amsterdam, Netherlands). Plant cuttings of chrysanthemum and apple mint were used for rooting, and the cuttings, including two nodes, were placed in a plug pot containing soil mix. The mixture was prepared with vermiculite (Verminuri, GFC Co., Ltd., Korea) and horticultural medium (Hanareum, Shinsung Mineral Co., Korea) in a ratio of 1:1 (v:v). Half of the cuttings were supplemented with 0.5 mL of BCS twice a week, while the others were supplemented only with water. After 3 weeks, the cuttings were pulled out of the plug pot, and the soil was washed from the roots to count the number of adventitious roots and measure the root mass.

To investigate its effects on early-stage plant growth, 10 rice (*Oryza sativa* subsp. japonica. ‘Koshihikari’) seeds were germinated in pots containing soil mix and supplemented with 1 mL BCS twice a week. The lengths of the longest leaves of each plant were measured for 4 weeks.

### Statistical analysis

The data are presented as the means ±standard deviations. To verify statistical significance, we performed analysis of variance (ANOVA) and Duncan’s multiple range test (DMRT) using R (4.0.2).

## Results

### Identification of the bacterial strain

**Fig. 1:**
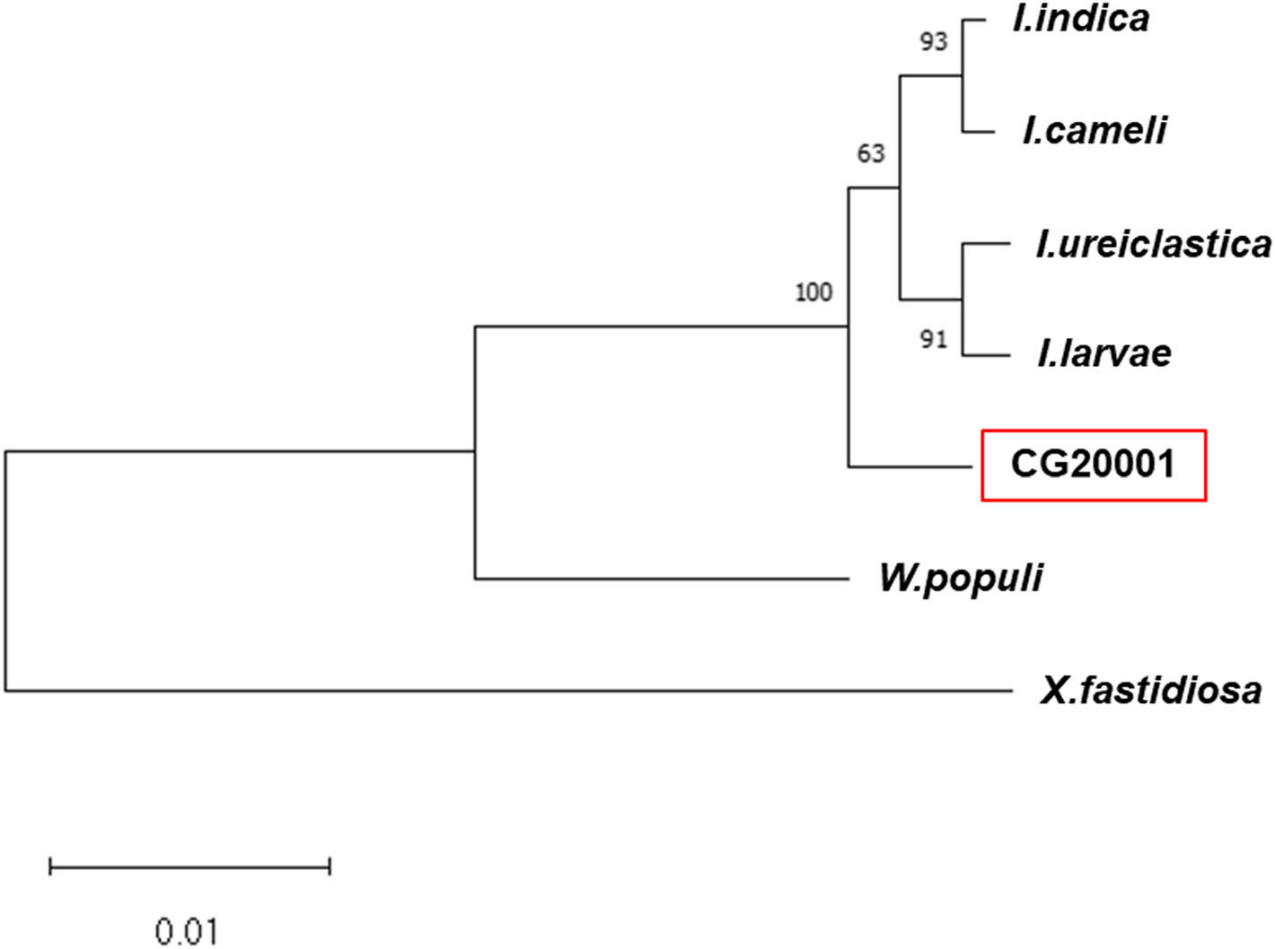
Phylogenetic tree constructed using the neighbor-joining method [21] based on 16S rDNA sequences. The bootstrap values of 500 replications are shown next to the branches [22]. The tree construction and bootstrap tests were conducted in MEGA X [23]. The GenBank IDs of the sequences are LC377575, MT758087, AS252143, MT759849, DQ991182, MT527537, and MZ389060 for *I. cameli*, *I. indica*, *I. larvae*, and *I. ureiclastica*, *Xylella fastidiosa*, *Wohlfahrtiimonas populi*, and *Ignatzschineria* sp. respectively

A Salkowski-stained clone was isolated and identified via 16S rDNA sequencing among the colonies isolated from the food process waste. The sequence showed the highest similarity with *Ignatzschineria* species in the NCBI database, with a few nucleotides differing from other *Ignatzschineria* species (Fig. S2). The next closest bacterial species were *Xylella* and *Wohlfahrtiimonas populi* within the database, with sequence identities lower than 90%. Therefore, we designated this newly isolated strain as *Ignatzschineria* sp. CG20001. The phylogenetic relationships among these strains are shown in Fig. 1. The 16S rRNA sequence of CG20001 was deposited in GenBank under the accession ID MZ389060.

### Confirmation of the presence of IAA in the BCS

**Fig. 2:**
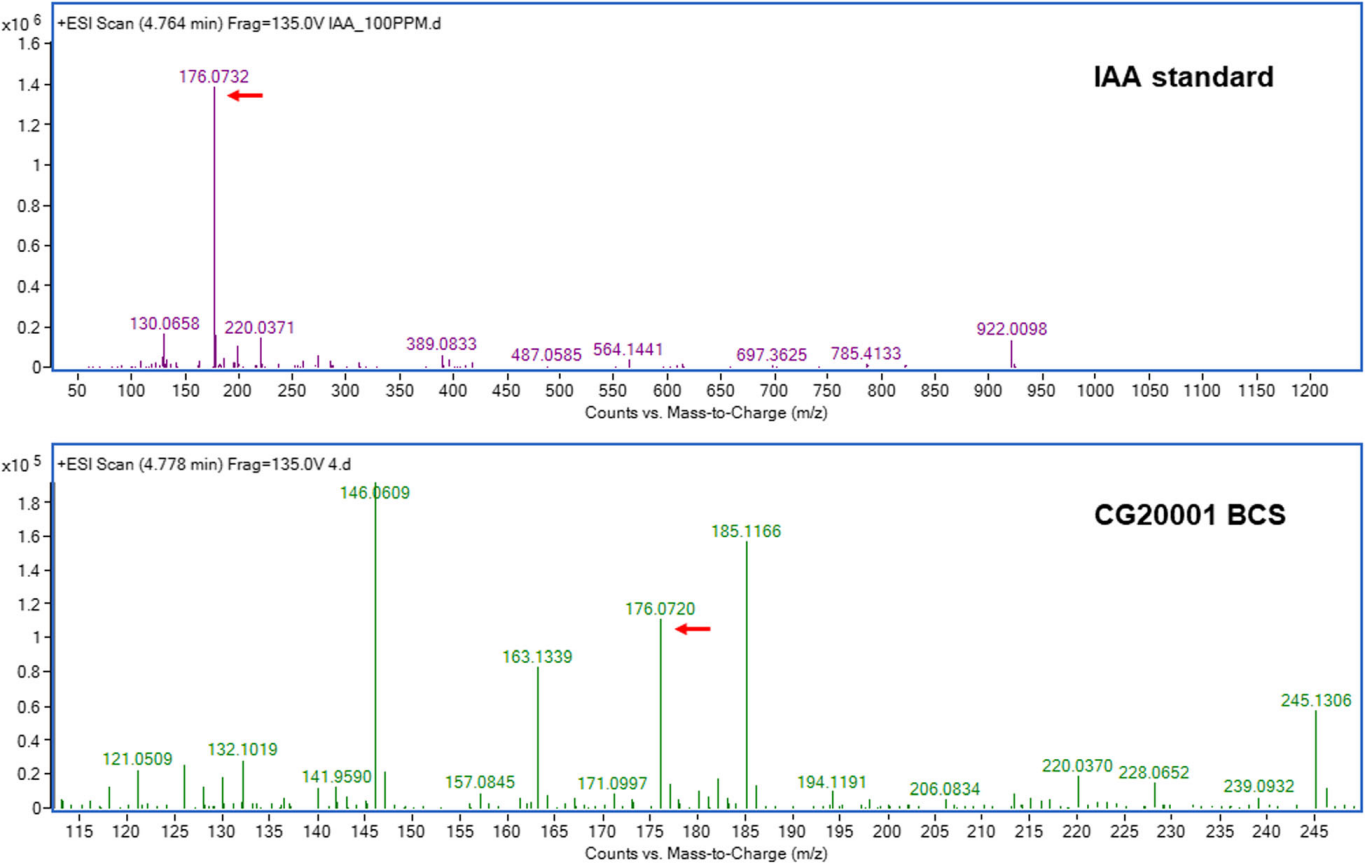
LC-MS/MS analysis of IAA in the CG20001 culture supernatant. LC-MS positive ion scan spectrum of free IAA (upper panel), and MS/MS spectra of BCS in the positive ESI mode (lower panel)

The BCS of the newly isolated strain was subjected to LC-MS/MS analysis for qualitative verification of IAA in the bacterial culture. One of the distinctive mass spectra was assigned to IAA based on the IAA standard solution. The molecular weight of free IAA with positive ionization resulted in a molecular ion at *m/z* 176. The ion at *m/z* 176.07 matched that of the IAA standard full spectrum (Fig. 2). These findings indicate that the substance formed by the *Ignatschineria* sp. CG20001 was, in fact, IAA.

### Effect of carbon source and precursor on IAA production

Since the strain was isolated from a material with high starch content, we briefly compared the effects of carbon sources on the growth and IAA production of the strain. The concentration of glucose had little effect on IAA productivity, and starch concentration was not correlated with IAA producibility either.

**Fig. 3:**
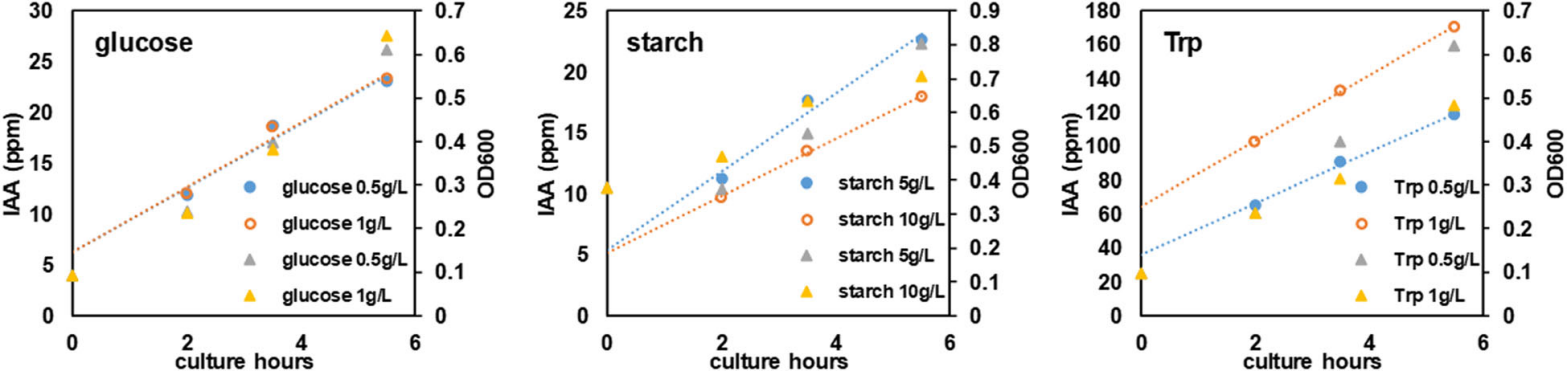
Effect of carbohydrates and precursor Trp supply on IAA production in *Ignatzschineria* sp. CG20001. Circles (open orange circles and solid light blue circles) with lines represent IAA concentrations in the BCS and triangles (yellow and gray) represent optical cell density (OD_600_)

In contrast, the precursor of IAA, Trp, had a significant impact on IAA productivity, showing higher IAA concentration in the BCS of the Trp-treated cultures. In the Trp-containing broth, IAA concentration increased from 102.5 to 170.5 mg/L within 3.5 h (Fig. 3).

Moreover, the UV absorption spectra clearly demonstrate that the basal growth medium does not include any Salkowski reagent reactive chemicals (Fig. S3).

### Inducibility of IAA production by the precursor Trp

**Fig. 4:**
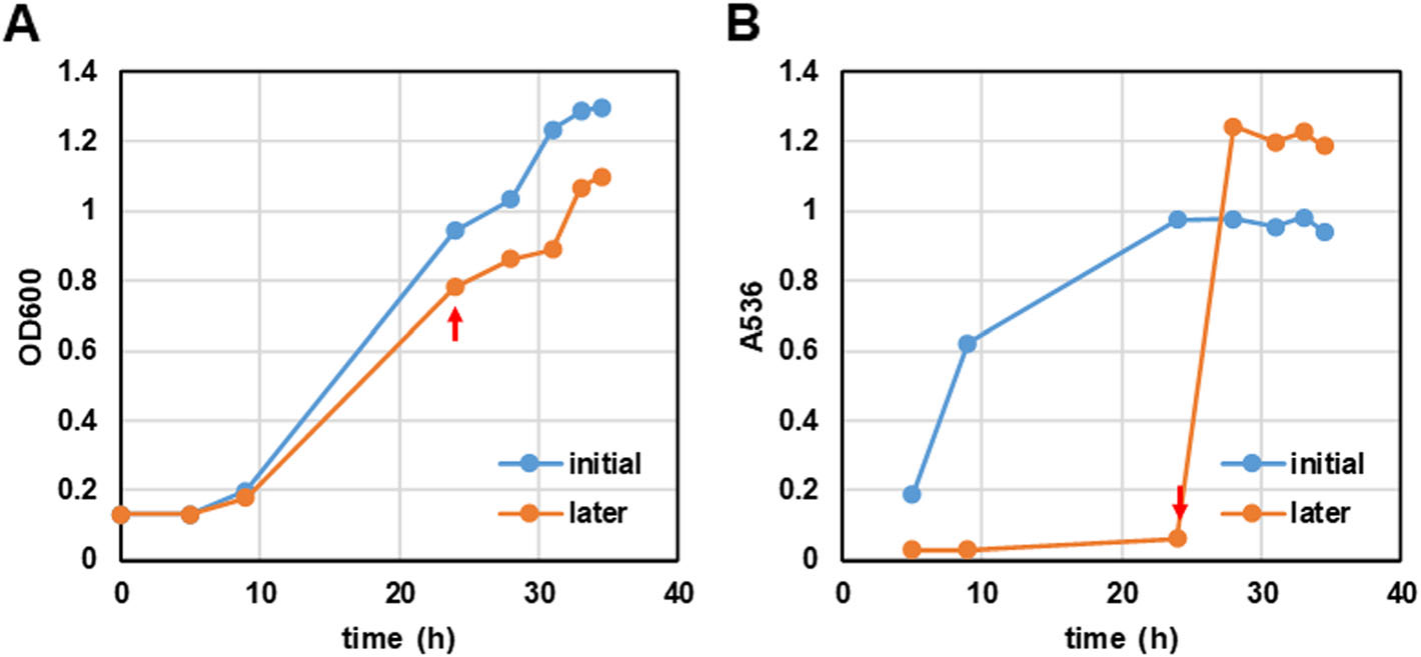
Inducibility of IAA production through the addition of l-tryptophan. The growth of CG20001 (**A**) and absorbance of Salkowski reaction of the culture (**B**). Trp was added at the start of cultivation (blue line), or at the marked time point (red arrows) in the orange line. Trp was added at a final concentration of 0.025%

To understand the IAA production kinetics in this strain, we added Trp at different growth stages, one at the start of culture and the other at the late exponential stage. The growth of CG20001 was almost identical whether Trp exists or not. However, IAA content in the culture increased with growth only in the Trp-supplemented culture. In contrast, IAA content was low without Trp addition even when it reached the mid-exponential stage; however, it markedly increased with the addition of Trp (Fig. 4). The results showed that IAA production in this strain was highly dependent on the presence of the precursor, Trp.

### Effect of the bacterial culture supernatant on rooting and plant growth

**Fig. 5:**
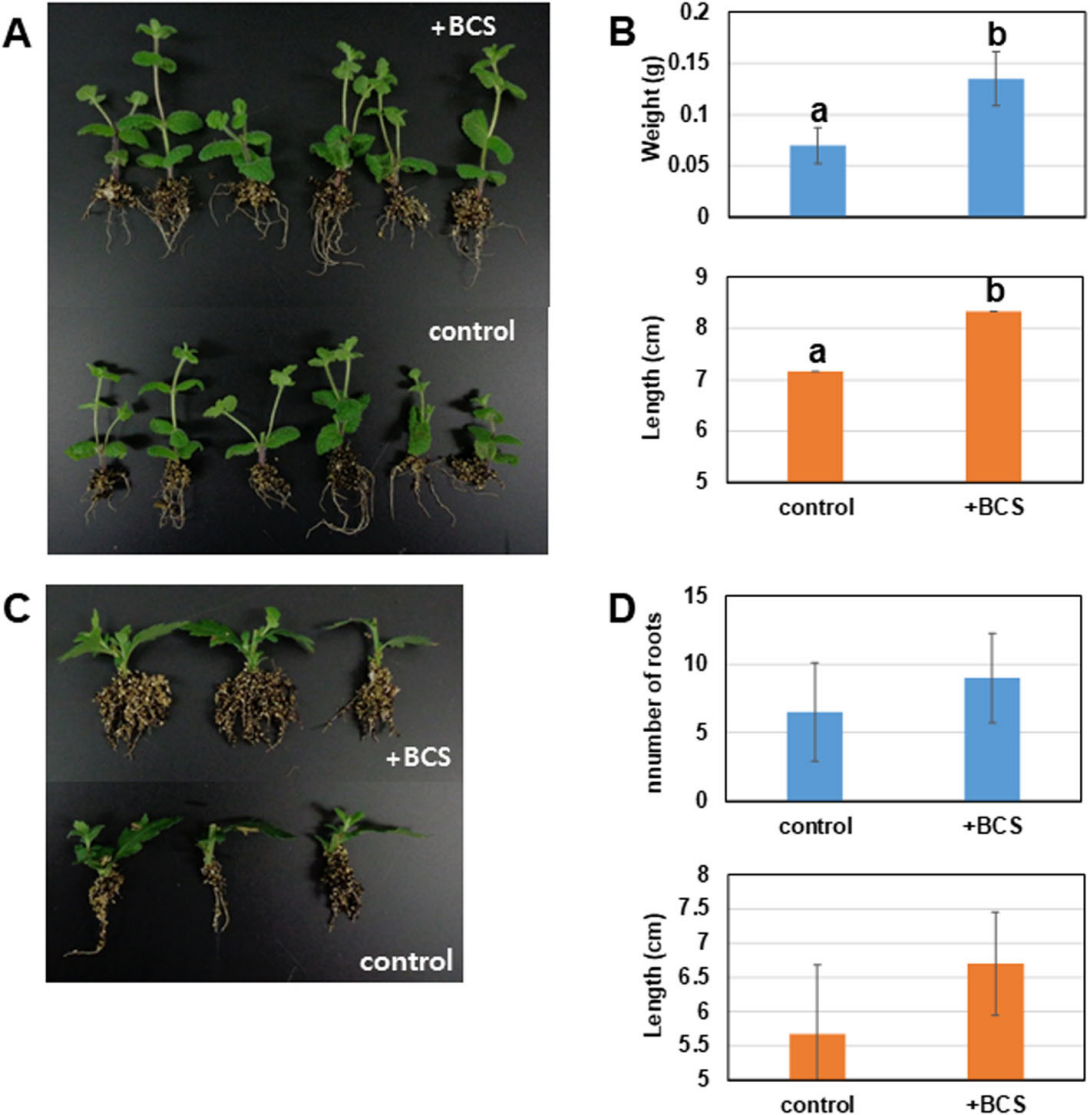
Effect of the BCS of *Ignatzschineria* sp. CG20001 on adventitious root formation in plant cuttings of apple mint (**A**, **B**) and chrysanthemum (**C**, **D**). The data represent the average obtained from six independent cuttings with the corresponding standard deviation. Bars with different letters indicate significant differences at *p* < 0.001

As the BCS contains a high amount of IAA, we investigated its effect on adventitious root formation in plant cuttings. The application of BCS to cuttings greatly improved root numbers and root masses in chrysanthemum and apple mint cuttings (Fig. 5). The average number and length of adventitious roots of chrysanthemum were also higher in BCS-treated cuttings although there was no significant difference. In apple mint cuttings, significantly compared to the control (*p* < 0.001), the average weight and length of adventitious roots were nearly twice as high in BCS-treated cuttings.The weight and total length of adventitious roots were 0.07 g and 7.16 cm in the untreated group, and 0.14 g and 8.33 cm in the BCS treatment, respectively.

**Fig. 6:**
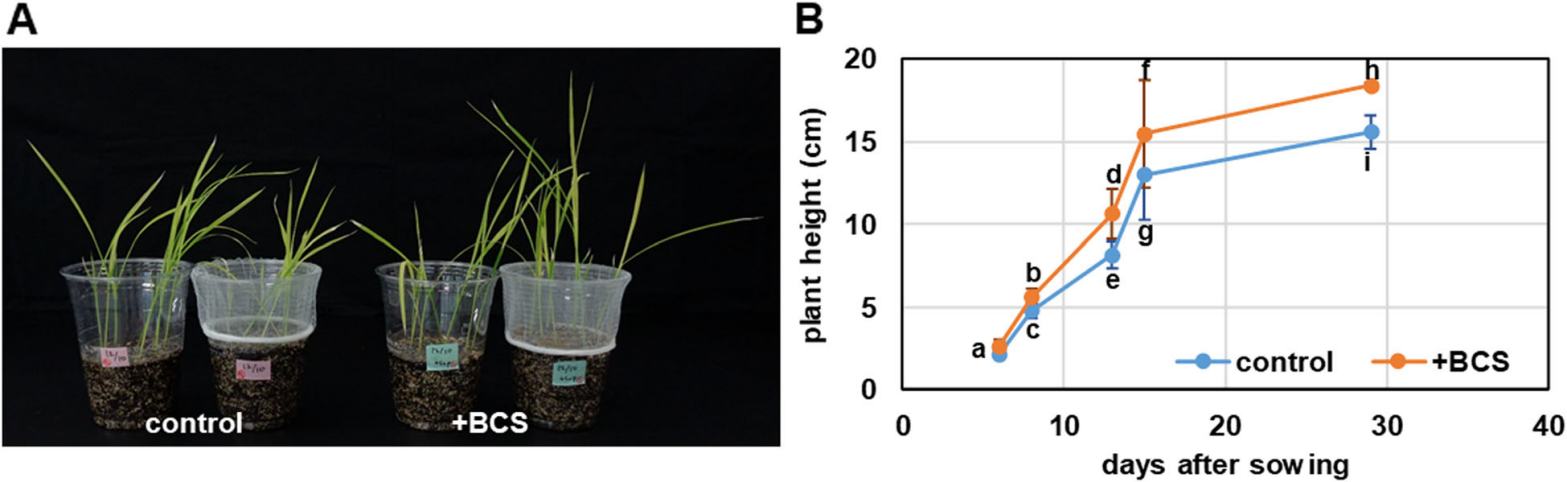
Effect of BCS treatment on the early growth of rice seedlings. Individual data points are expressed as mean ± SE (*n* = 10) of control shoot growth on soil mix with BCS or not. For each graph, different lowercase letters indicate a significant difference (*p* < 0.05)

Better root growth can positively influence plant growth; therefore, we compared the growth of rice seedlings in the early stages. Treatment with BCS enhanced the growth of rice plants within the first month (Fig. 6).

## Discussion

In this study, we isolated an IAA-producing bacterium from food process waste through the entire composting process using numerous screening schemes. Then, we characterized its IAA productivity and effects of BCS on plant root and seedling growth, despite the statistical limitations of our experiment using a small number of individuals with similar growth patterns among several individuals.

The characteristics of IAA production by this strain are Trp-dependent, and the addition of glucose and starch had no effect on IAA productivity. The effects of carbon sources on IAA production vary from strain to strain and, in some cases, also affect auxin production through bacterial growth caused by different sugar utilization preferences among bacteria [28–30]. Nitrogen sources also affect bacterial IAA production. Blinkov et al. (2014) reported the different effects of nitrate and ammonium as inorganic nitrogen sources for IAA production in *Klebsiella planticola* strain TSKhA-91 [17]. Different kinds of organic nitrogen also affected bacterial auxin production in different ways. Another auxin-producing bacterium, isolated by Chandra et al. [18], was reported to produce IAA most efficiently by supplying dextrose as a carbon source and beef extract as a nitrogen source. They did not identify the strain isolated; however, these results show the diverse mechanisms that can induce efficient IAA production in different bacteria. Di et al. [16] described various pathways of IAA production in different species, since diverse evolutionary paths of IAA biosynthesis can be predicted in both plants and their interacting bacteria. Unlike nutritional effects, the effect of Trp is critical in almost all IAA-producing bacterial strains. In the most well-known pathway of the five IAA biosynthetic pathways, Trp is converted to indole-3-acetamide by tryptophan monooxygenase (encoded by the iaaM gene) and then catalyzed into IAA by indole acetamide hydrolase (encoded by the iaaH gene) [31, 32]. Three other pathways were also found to be Trp-dependent. Trp-independent pathways have been reported to exist in plants as a measure of maintaining a basal level of auxin [33]; however, there are few reports on such pathways in bacterial IAA biosynthesis. Moreover, even in plants, the Trp-independent pathway has not been clearly elucidated [16]. In bacteria, IAA production usually depends on the presence of Trp; however, the optimal concentration and maximum productivity vary according to the species.

The root growth-promoting effects of auxin-producing bacteria have been reported in many previous reports [6, 10, 34]. The robust growth of roots improves plant fitness due to enhanced water and nutrient uptake through the root from an early growth stage. These growth-promoting effects have also been reported in other studies [27, 35, 36]. Blinskov et al. (2014) also emphasized the importance of rapid growth in some areas of high latitude where the arable period is short, representing the effect of *Klebsiella planticola* TSKhA-91 strain, which induced rapid germination and growth of cucumber seedling under cold stress [17].

It is interesting to note that the whole genome of *Ignatzschineria cameli* was sequenced and reported by Tsang et al. [37]; however, we could not find IAA biosynthesis-related genes in the annotation table or conserved domain sequence in the whole genome sequence. However, it does not remove the possibility for CG20001 to harbor IAA biosynthesis-related genes in its genome, because they also pointed out that the strain *I. cameli* is distinctive from other *Ignatzschineria* strains both in their gene sequences and phenotypes. There have been no previous reports on *Ignatzschineria* species that interact with plant roots or are present in the rhizosphere. Therefore, it is unusual for this bacterium to produce IAA with such high efficiency. On the other hand, many enteric bacteria have been found to reside both on the surfaces of plants and animal intestines [38, 39]. Cox et al. suggested that enteric pathogens producing IAA may use plants as alternative hosts [15]. Considering that some *Ignatzschineria* species are found in the intestines of maggots in addition to various environmental sources [40], it is a reasonable hypothesis that CG20001 may also use plants as an alternative host.

In terms of the effect of bacterially produced auxin, IAA does not only trigger root growth, but also many different physiological signals in plants, including secondary metabolism [41] and various developmental stages [42]. The IAA signal finally affects almost every aspect of plant physiology, including stress responses [43–45] and interactions with pathogens [45, 46], through its intricate interconnection with other plant hormones [47–49]. Therefore, we have not yet clearly understood the IAA biosynthetic pathway, and the ecological advantage of this strain for producing auxin in theirs. However, the beneficial effects of the culture of this strain are presented in this study, demonstrating the potential of this strain for agricultural applications.

## Conclusions

Bacterial traits that promote plant root growth are advantageous, making them important agricultural tools. These important traits found in various microbes, including both symbiotic and pathogenic bacteria in plants, function through signals of the plant hormone auxin. The auxin-producing strain we reported herein shows high efficiency of IAA production, relatively higher than that of previously reported strains. The strain in this study produced 16.6 mg/L IAA every hour, which was much more efficient than the yield of 104 mg/L for 36 h reported by Chandra et al. [18] and 109.9 mg/L for 24 h in *Enterobacter aerogenes* reported by Oh et al. [46]. Consequently, this bacterium could be potentially utilized in agriculture and as a valuable genetic resource due to its novelty and efficiency in IAA production.

## Supplementary Information

**Additional file 1: Fig. S1.** Tryptophan-dependent biosynthetic pathways of IAA in plants and bacteria. The soild black lines are common in plants and bacteria. The pathway is simplified to compare that of plants and bacteria and is therefore drawn around the steps that shared by both. The steps represented in blue lines are present in bacteria, and while those in green lines are present in plants. The genes involved in the dashed lines has not been identified.

**Additional file 2: Fig. S2.** Alignment of 16S rRNA sequences from *Ignatzschineria* species. The GenBank IDs of the sequences used for the multiple alignment are LC377575, MT758087, AS252143, and MT759849 for *I. cameli*, *I. indica*, *I. larvae*, and *I. ureiclastica*, respectively.

**Additional file 3: Fig. S3.** Colors of the Salkowski reactions (A, B) and their absorption spectra (C, D). IAA was added to final concentrations of 25, 50 mg/L and Trp were added to final concentrations of 0.05, 0.1, and 0.2% (A), and the corresponding absorption spectra are shown in (C). Either Trp (0.05%) or Trp in combination with IAA (50 mg/L) were added to LB broth, and the Salkowski reaction (B) and absorption spectra (D) are shown.

## Data Availability

Not applicable

## Abbreviations:

PRA: Intermediates
CdRP: phosphoribosyl anthrarnilate
IGP: 1-(o-carboxyphenylamino)-1-deoxyribulose 5-phosphate
TAM: indole-3-glycerol phosphate
IAOx: tryptamine
IDA: indole-e-acetaldoxime
IAN: indole-3-acetaldehyde
IAM: indole-3-acetonitrile
IPyA: indole-3-acetamide
IAD: indole-3-pyruvic acid indole-3-acetaldehyde
ASA1/ASB1: Enzymes:anthranilate synthase α/β
PAT1: prephenate aminotransferase
PAI1/2/3: phosphoribosylanthranilate isomerase
IGS: indole-3-glycerol phosphate synthase
TSA1: Trp synthase α
TSB: Trp synthase β
TDC: Trp decarboxylase
AO: aldehyde oxidase
AT: amino transferase
Oxd: indolacetaldoxime dehydratase
YUCCA: Trp decarboxylase/Flavin monooxygenase-like protein
AMI1: Amidase iaaH, Trp hydrolase
NIT: nitrilase; iaaM, Trp monooxugenase
TAA1: Tryptophan aminotransferase of *Arabidopsis* 1
CYP79B2/B3: Trp-specific P450 monooxygenase
AAO: aldehyde oxidase
