# Supplementary figures and images for: A highly efficient auxin-producing bacterial strain and its effect on plant growth
Source: J Genet Eng Biotechnol. 2021 Dec 2;19:179. doi: 10.1186/s43141-021-00252-w (PMC8639878; doi:10.1186/s43141-021-00252-w)

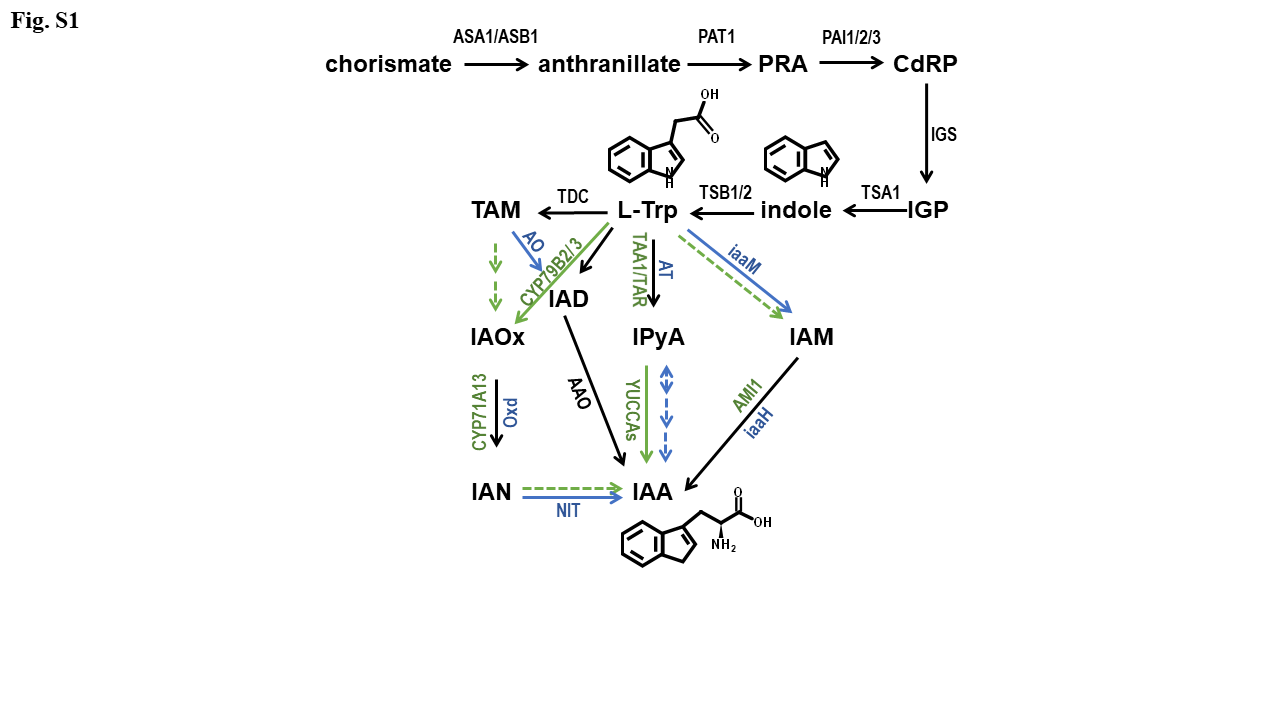

Supplement: Supplementary file 1 — Additional file 1: Fig. S1. Tryptophan-dependent biosynthetic pathways of IAA in plants and bacteria. The soild black lines are common in plants and bacteria. The pathway is simplified to compare that of plants and bacteria and is therefore drawn around the steps that shared by both. The steps represented in blue lines are present in bacteria, and while those in green lines are present in plants. The genes involved in the dashed lines has not been identified. [file 43141_2021_252_MOESM1_ESM.tif]

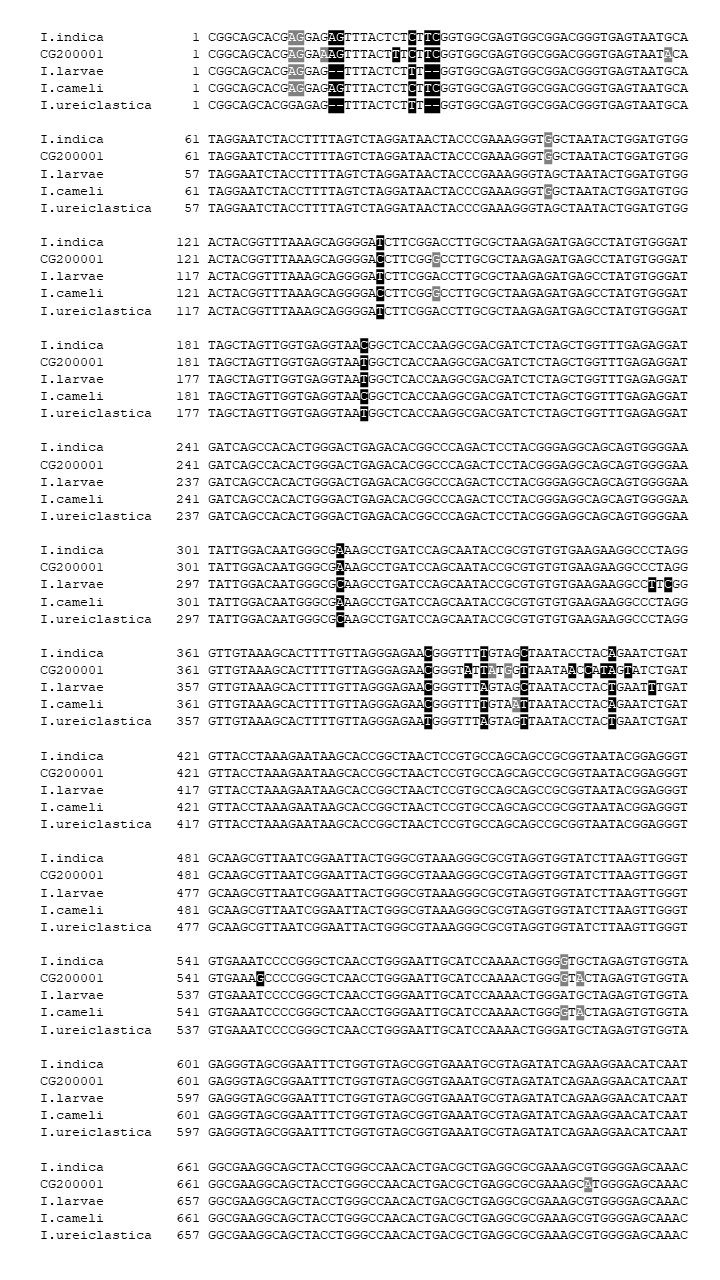

Supplement: Supplementary file 2 — Additional file 2: Fig. S2. Alignment of 16S rRNA sequences from Ignatzschineria species. The GenBank IDs of the sequences used for the multiple alignment are LC377575, MT758087, AS252143, and MT759849 for I. cameli, I. indica, I. larvae, and I. ureiclastica, respectively. [file 43141_2021_252_MOESM2_ESM.zip › Supplemental figure 2-1R3.TIF]

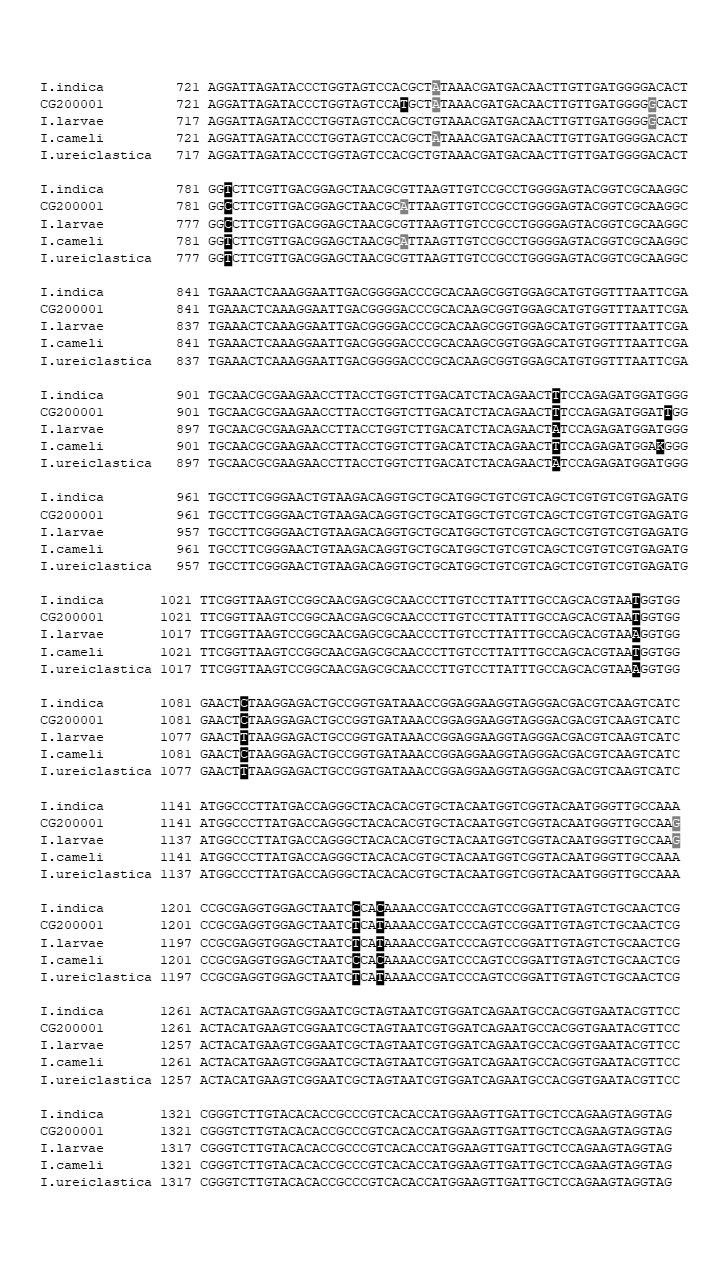

Supplement: Supplementary file 2 — Additional file 2: Fig. S2. Alignment of 16S rRNA sequences from Ignatzschineria species. The GenBank IDs of the sequences used for the multiple alignment are LC377575, MT758087, AS252143, and MT759849 for I. cameli, I. indica, I. larvae, and I. ureiclastica, respectively. [file 43141_2021_252_MOESM2_ESM.zip › Supplemental figure 2-2R3.TIF]

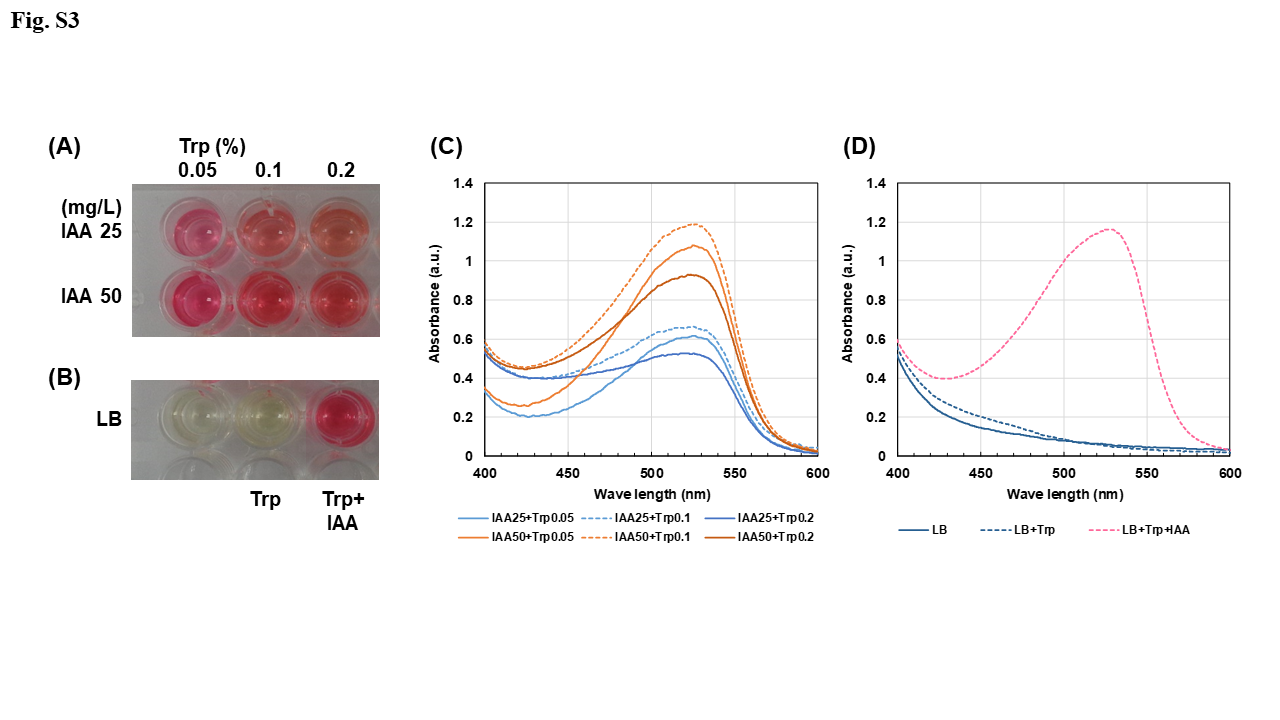

Supplement: Supplementary file 3 — Additional file 3: Fig. S3. Colors of the Salkowski reactions (A, B) and their absorption spectra (C, D). IAA was added to final concentrations of 25, 50 mg/L and Trp were added to final concentrations of 0.05, 0.1, and 0.2% (A), and the corresponding absorption spectra are shown in (C). Either Trp (0.05%) or Trp in combination with IAA (50 mg/L) were added to LB broth, and the Salkowski reaction (B) and absorption spectra (D) are shown. [file 43141_2021_252_MOESM3_ESM.tif]
